# Clinical Phenotypes of a Pediatric Cohort with *GDF2*-Related Hereditary Hemorrhagic Telangiectasia

**DOI:** 10.3390/jcm14103359

**Published:** 2025-05-12

**Authors:** Owen Oliver, Allison D. Britt, Alexandra J. Borst, Elizabeth Goldmuntz, Nihal Bakeer, Shih-shan Lang, Stephanie Fuller, Arastoo Vossough, Lauren A. Beslow

**Affiliations:** 1 Division of Neurology, Children’s Hospital of Philadelphia, 3401 Civic Center Blvd, Philadelphia, PA 19104, USA; oweno2025@gmail.com; 2 Comprehensive Vascular Anomalies Program, Children’s Hospital of Philadelphia, 3401 Civic Center Blvd, Philadelphia, PA 19104, USA; brittad@chop.edu (A.D.B.); alexborst@med.unc.edu (A.J.B.); bakeern@chop.edu (N.B.); 3 Division of Hematology, Children’s Hospital of Philadelphia, 3401 Civic Center Blvd, Philadelphia, PA 19104, USA; 4 Department of Pediatrics, Perelman School of Medicine at the University of Pennsylvania, 3400 Civic Center Blvd, Philadelphia, PA 19104, USA; goldmuntz@chop.edu; 5 Division of Cardiology, Children’s Hospital of Philadelphia, 3401 Civic Center Blvd, Philadelphia, PA 19104, USA; 6 Division of Neurosurgery, Children’s Hospital of Philadelphia, 3401 Civic Center Blvd, Philadelphia, PA 19104, USA; chens4@chop.edu; 7 Department of Neurosurgery, Perelman School of Medicine at the University of Pennsylvania, 3400 Civic Center Blvd, Philadelphia, PA 19104, USA; 8 Division of Cardiac Surgery, Children’s Hospital of Philadelphia, 3401 Civic Center Blvd, Philadelphia, PA 19104, USA; fullers@chop.edu; 9 Department of Cardiothoracic Surgery, Perelman School of Medicine at the University of Pennsylvania, 3400 Civic Center Blvd, Philadelphia, PA 19104, USA; 10 Division of Neuroradiology, Children’s Hospital of Philadelphia, 3401 Civic Center Blvd, Philadelphia, PA 19104, USA; 11 Department of Radiology, Perelman School of Medicine at the University of Pennsylvania, 3400 Civic Center Blvd, Philadelphia, PA 19104, USA; 12 Department of Neurology, Perelman School of Medicine at the University of Pennsylvania, 3400 Civic Center Blvd, Philadelphia, PA 19104, USA

**Keywords:** *GDF2*, hereditary hemorrhagic telangiectasia, nosebleeds, epistaxis, arteriovenous malformation, pulmonary hypertension

## Abstract

**Background/Objectives:** Pathogenic variants in the growth differentiation factor 2 (*GDF2*) gene have been linked to a hereditary hemorrhagic telangiectasia (HHT)-like syndrome, yet their clinical significance remains under investigation. This study reports seven pediatric patients with *GDF2* variants from a single center. **Methods:** We identified children with *GDF2* pathogenic variants and variants of uncertain significance (VUS) from the Children’s Hospital of Philadelphia Comprehensive HHT Program and cross-referenced the list with a full-text query by *GDF2* gene name on >53,000,000 visits to ensure complete ascertainment. Medical records were reviewed retrospectively, and variables of interest were abstracted. **Results:** The median age at genetic testing was 12 years (range 1.75–16). Reasons for genetic testing included telangiectasias, pulmonary hypertension, familial testing, respiratory symptoms, seizures, developmental disabilities, and lung arteriovenous malformations (AVMs). Four patients had missense VUS, including two novel VUS (c.34C>G; p.Leu12Val, c.41C>T; p.Ser14Phe), while three had pathogenic deletions. All patients experienced epistaxis, starting at a median age of 6 years (range 2–12). Three had telangiectasias. One patient had both a *GDF2* VUS and a *de novo* partial endoglin (*ENG*) gene deletion. While this patient’s symptoms of HHT are likely related to her *ENG* variant, synergy cannot be excluded, and two first-degree family members with clinically significant epistaxis also have the same *GDF2* VUS. Notably, two patients had visceral AVMs—one with a lung AVM and another with a vein of Galen malformation. **Conclusions:** Interpretation of *GDF2* VUS and their relationship to clinical symptoms is challenging given the rarity of these genetic variants and the inadequate diagnostic utility of the current clinical criteria for HHT in the pediatric population. Further research with larger cohorts is necessary to improve the genotype–phenotype correlation in *GDF2*-related HHT. Carefully collected clinical information with longitudinal follow-up may also assist in refining classification of *GDF2* VUS as benign or pathogenic in the future.

## 1. Introduction

Hereditary hemorrhagic telangiectasia (HHT), also known as Osler–Weber–Rendu syndrome, is an inherited autosomal dominant vascular dysplasia characterized by malformed blood vessels that can pose life-threatening risks. Clinical diagnosis is defined by the Curaçao criteria, which include recurrent epistaxis, telangiectasias at characteristic sites (oral cavity, fingers, nose, gastrointestinal tract), organ arteriovenous malformations (AVMs), and the diagnosis of a first-degree family member. Arteriovenous shunting associated with AVMs can lead to a variety of complications, including stroke, hemorrhage, hepatic encephalopathy, high-output cardiac failure, and portal or pulmonary hypertension [1,2,3,4,5,6,7].

Around 1 in 5000 to 10,000 people is affected by HHT. A genetic cause has been identified in ~85% of clinically diagnosed HHT patients [3]. HHT pathogenesis is due to abnormal angiogenesis related to genetic variants in the transforming growth factor-beta (TGF-β) pathway. The TGF-β pathway regulates cell proliferation, mesodermal differentiation, apoptosis, and cell migration. There are three primary genes associated with HHT: (1) endoglin (*ENG*) which encodes the endoglin protein, a non-intracellular co-receptor, (2) activin A receptor-like type 1 (*ACVRL1*) which encodes the activin receptor-like kinase-1 (ALK1), a serine–threonine intracellular receptor, and (3) suppressor of mothers against decapentaplegic (*SMAD4*) an intracellular transcription factor protein that aids transduction from the endoglin and ALK1 proteins toward gene expression.

A more recent discovery is the pathogenesis of HHT through alterations in bone morphogenetic protein 9 (BMP9), encoded by growth differentiation factor 2 (*GDF2*). BMP9 is an activating ligand within the TGF-β pathway, which signals for vascular quiescence during angiogenesis [8,9]. Fewer than 1% of individuals with clinical HHT have a pathogenic *GDF2* variant [3,10,11,12], which causes *GDF2*-related HHT [3,10,11,13]. There are few reports of *GDF2*-related HHT in the literature, therefore the full phenotypic spectrum of patients with *GDF2* variants is unknown [11,12,14,15,16,17,18,19]. This report aims to illustrate phenotypic findings of individuals with pathogenic variants and variants of uncertain significance (VUS) in *GDF2* within a pediatric cohort to fill these gaps in knowledge and expand the understanding of clinical manifestations of *GDF2*-related HHT.

## 2. Materials and Methods

We identified children with *GDF2* pathogenic variants and VUS from the Children’s Hospital of Philadelphia (CHOP) Comprehensive HHT Program diagnosed between 2019 and 2024 and cross-referenced the list with a full-text query by *GDF2* gene name on >53,000,000 visits in the institutional electronic health record (EHR) database to ensure complete ascertainment. Clinical, imaging, and genetic testing reports were reviewed from the EHR. Genetic testing included the following: Invitae (San Francisco, CA, USA) six gene panel (*ACVRL1*, *ENG*, *EPHB4*, *GDF2*, *RASA1*, *SMAD4*) for patients 1 and 6, Invitae 14 gene pulmonary arterial hypertension panel (*ACVRL1*, *AQP1*, *ATP13A3*, *BMPR1B*, *BMPR2*, *CAV1*, *EIF2AK4*, *ENG*, *GDF2*, *KCNA5*, *KCNK3*, *SMAD9*, *SOX17*, *TBX4*) for patient 2 (patient 3 with familial directed testing), CHOP Division of Genomic Diagnostics (Philadelphia, PA, USA) 67 gene comprehensive pulmonary-vascular sequencing and deletion/duplication panel including (*ACVRL1*, *ENG*, *GDF2*, *RASA1*, *SMAD4*) for patient 4, genome-wide single nucleotide polymorphism microarray analysis from the CHOP Department of Pathology and Laboratory Medicine (Philadelphia, PA, USA) and exome sequencing from GeneDx (Gaithursburg, MD, USA) for patient 5, and ARUP Laboratories (Salt Lake City, UT, USA) 6 gene hereditary hemorrhagic telangiectasia sequencing and deletion/duplication panel (*ACVRL1*, *ENG*, *EPHB4*, *GDF2*, *RASA1*, *SMAD4*) for patient 7. We then performed a literature review and compared our patients to those in seven previous publications. The Institutional Review Board approved the study and waived informed consent and assent.

## 3. Results

**Figure 1:**
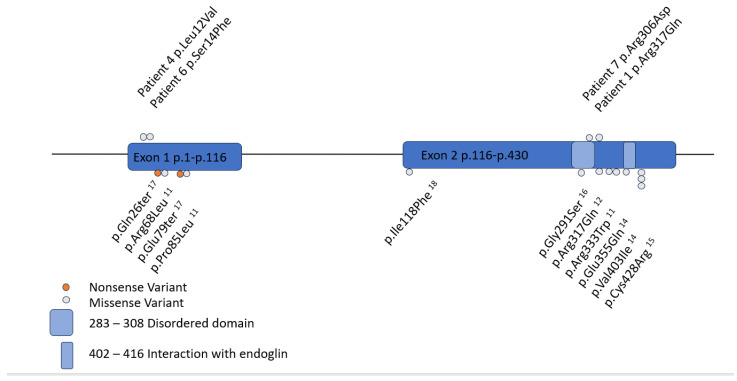
*GDF2* gene map. Exons 1 and 2 indicated by blue boxes. Important domains indicated by the light blue boxes. Variants in CHOP patients are indicated by gray dots on the top. Variants found in the literature indicated by gray and orange dots on the bottom. Gray indicates missense, orange indicates nonsense variants. Of note, three CHOP patients with full gene deletions and two literature patients with full gene deletions are not included in this figure.

**Figure 2:**
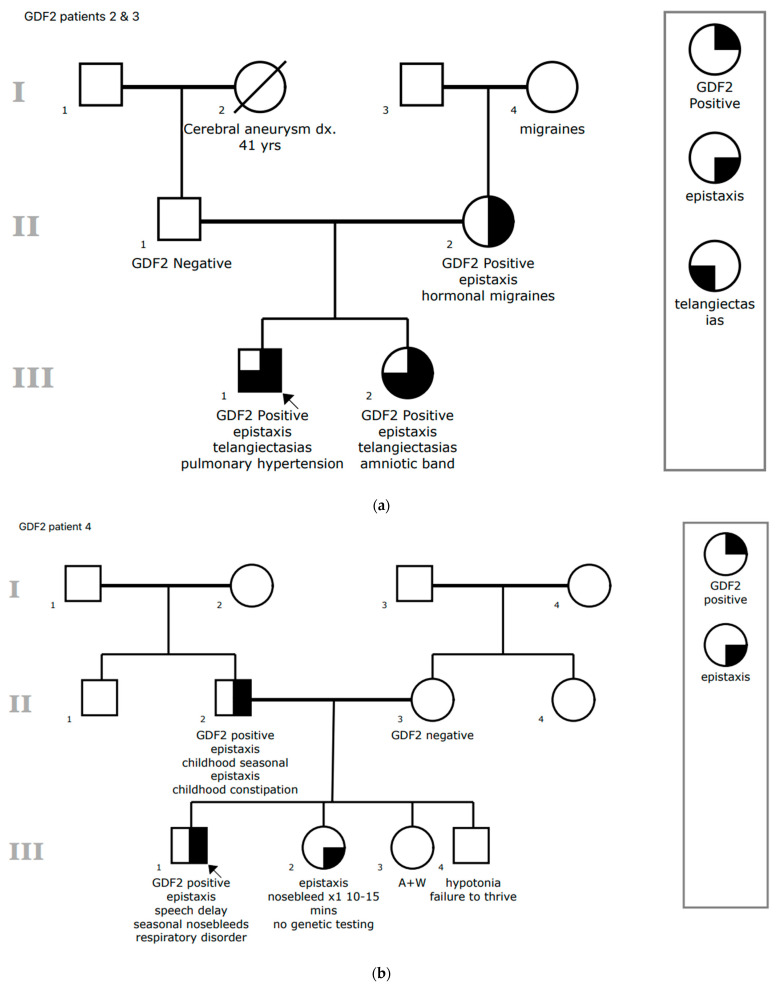

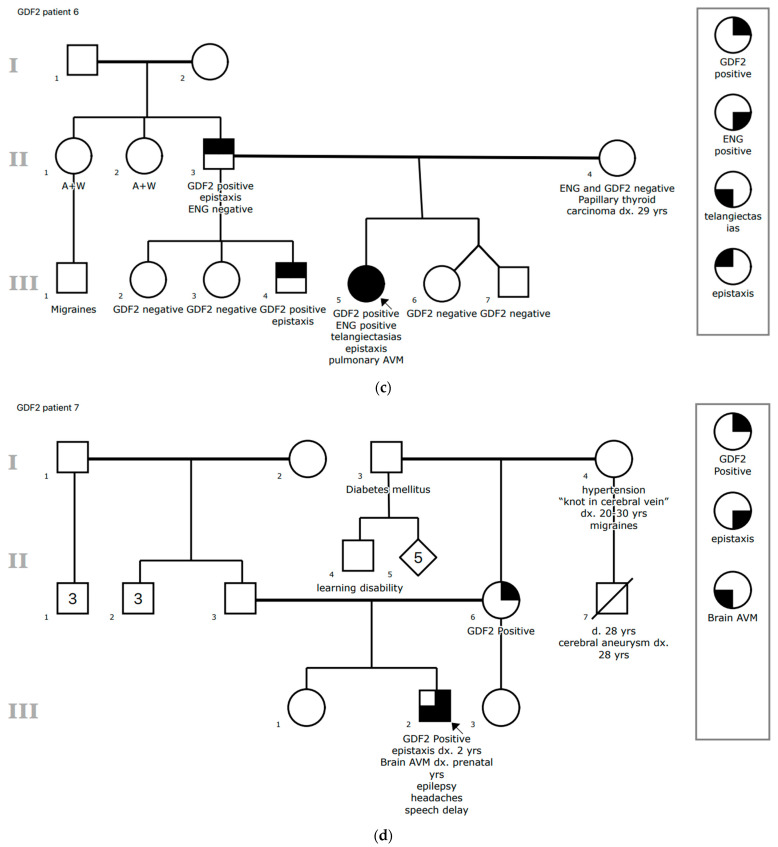
Pedigrees of 5/7 patients with *GDF2* variants. (**a**) Pedigree of patients 2 and 3 showing maternally inherited *GDF2* VUS. (**b**) Pedigree of patient 4 showing paternally inherited *GDF2* VUS. Father (II, 2) and sister (III, 2) have epistaxis. (**c**) Pedigree of patient 6 showing proband with *de novo ENG* and paternally inherited *GDF2* VUS. Epistaxis in father (II, 3) and paternal half-brother (III, 4) tracks with the *GDF2* VUS. (**d**) Pedigree of patient 7 showing the possible cerebral vascular malformation in maternal grandmother (I, 4) and cerebral aneurysm at age 28 in maternal first cousin once removed (II, 7). No family member cascade testing has been completed, but the maternal grandmother with a “knot” in the cerebral veins could be relevant if the variant is found to be inherited from the maternal side.

Seven patients with *GDF2* variants were evaluated in the CHOP Comprehensive HHT Program between 2019 and 2024 (Table 1). No other patients with *GDF2* variants were identified through the full-text EHR query. Four of the seven patients were male. Median age at genetic testing was 12 years (range 1.75–16). Four patients had *GDF2* missense VUS, and three had full gene deletions of *GDF2* (Figure 1). All seven patients had epistaxis at a median age of 6 years of age (range 2–12 years), and three of seven patients had telangiectasias. Two of these developed additional telangiectasias over time. A fourth patient developed a single telangiectasia at two-year follow-up. All telangiectasias were typical in appearance for HHT, and no child had “spider telangiectasias.” Pedigrees are found in Figure 2 for the five patients for whom family history was known.

### 3.1. Case Series

#### 3.1.1. Patient 1

Eleven-year-old female who presented with seven small cutaneous telangiectasias across the right thumb (dorsal surface near metacarpal bone), upper arm, right upper lip, and the sole of the right foot. These appeared at the age of 3 years. A six gene HHT panel revealed a heterozygous missense variant in the *GDF2* gene (c.950G>A, p.Arg317Gln) which was classified as a VUS. The gnomAD allele frequency is 0.00005205, and there are 3 ClinVar submissions that classify this variant as a VUS. In silico analysis supports that this missense variant does not alter protein structure/function. This VUS has been previously reported in an individual with suspected HHT [12], and there are no functional studies for this variant.

She had nosebleeds which started at the age of 9 years and occurred monthly but did not require treatment. The patient was adopted, and biological parents were unavailable; thus, family history could not be obtained. She also experienced 1–2 mild frontal headaches per month. Brain magnetic resonance imaging (MRI) with and without contrast with accompanying head magnetic resonance angiogram (MRA) did not reveal brain vascular malformations. Oxygen saturation was 98% on room air, and contrast echocardiogram was negative for intrapulmonary shunting. At a follow-up visit 3 years after her initial HHT clinic visit, the patient had weekly nosebleeds requiring tranexamic acid, a new telangiectasia on the right knee, and heavy menses requiring initiation of an oral contraceptive pill.

#### 3.1.2. Patient 2

Eight-year-old male who presented after being diagnosed with pulmonary hypertension. Subsequent 14 gene pulmonary arterial hypertension panel revealed a pathogenic deletion of the entire coding sequence of *GDF2* as well as a VUS in bone morphogenetic protein receptor type 1B (*BMPR1B*). The boundaries of the deletion are unknown as they extend beyond the assayed region for *GDF2* and may include additional genes. This deletion was classified as pathogenic based on a previous report of two individuals with a full gene deletion of *GDF2* with pulmonary arterial hypertension but notably no clinical features of HHT [20]. Two individuals with *GDF2* gene deletions and clinical features of HHT have also been reported in the literature [14]. Pulmonary hypertension presented as syncopal episodes and headaches at six years of age. Further clinical history revealed frequent nosebleeds, occurring multiple times per week also since the age of six years, which were managed with nose clips and plugs. He did not have telangiectasias and contrast echocardiogram did not reveal evidence of lung AVMs. Most recent oxygen saturation was 97% on room air. Brain MRI with and without contrast and MRA head did not reveal brain vascular malformations. Due to this novel diagnosis, his relatives were screened, leading to the diagnosis of his mother and 13-year-old sister (patient 3) with the same pathogenic deletion of the *GDF2* coding sequence (Figure 2a). Patient 2 and 3’s mother did not have telangiectasias but experiences 1–2 nosebleeds per year. Patient 2 developed a single telangiectasia on the right fifth digit knuckle at his two-year follow-up.

#### 3.1.3. Patient 3

Thirteen-year-old female presented after her brother was diagnosed with a *GDF2* variant (patient 2). Known familial variant testing was positive for the familial deletion of the entire coding sequence. Her history was notable for infrequent nosebleeds since the age of six years. Clinical examination revealed telangiectasias on both hands (1 on dorsal hand, 2 on thumb) and right thigh (1) and truncation of the left distal foot and abnormal digits due to presumed amniotic band syndrome in utero. At follow-up 1 year later, she had nosebleeds approximately four to five times per year, and she had developed three new telangiectasias, two on the anterior surface of her right forearm and one on the anterior surface of her left forearm. A contrast echocardiogram was negative for intrapulmonary shunting, but a dysplastic pulmonary valve was noted. Brain MRI with and without contrast and MRA head did not reveal brain vascular malformations.

#### 3.1.4. Patient 4

Fifteen-year-old male who presented at age nine years with respiratory symptoms including asthma and a pneumothorax was evaluated for possible primary ciliary dyskinesia. A 67 gene comprehensive respiratory and vascular sequencing and deletion/duplication panel revealed a heterozygous missense VUS in *GDF2* (c.34C>G, p.Leu12Val). At the time of this genetic report, it was novel. In the 5 years since the report was issued, additional information about the variant has been reported, but it has not been reclassified. The gnomAD allele frequency is 0.000001890, and ClinVar contains one submission classifying this variant as a VUS. In silico predictions show SIFT: “Not Available”; PolyPhen-2: “Benign”; Align-GVGD: “Not Available”. This variant has not been previously reported in the literature, and there are no functional studies.

He had a history of infrequent nosebleeds concurrent with allergies starting at age 12 years that did not require intervention. No telangiectasias were identified. Oxygen saturation on room air was 99%. A CT chest to evaluate for bronchiectasis did not reveal evidence of lung AVMs, but contrast was not given. The patient and family declined brain imaging. Parental genetic testing identified that this VUS was inherited from the patient’s father who had nosebleeds as a child (Figure 2b).

#### 3.1.5. Patient 5 

Sixteen-year-old male who presented at age 4 years for possible seizures, developmental disabilities, and behavioral problems. A chromosomal microarray revealed a multigene deletion at chromosome 10q11.23, including the *GDF2* gene. At that time, the *GDF2* gene was not yet associated with HHT. Twelve years later, at age 16 years, he returned to genetics clinic, and the deleted region was queried again and found to include the *GDF2* gene, consistent with a diagnosis of HHT5. While haploinsufficiency/loss of function has not been definitively established as a mechanism of disease in *GDF2*-related HHT, two individuals with deletions including *GDF2* and clinical features of HHT have been reported in the literature [14]. Exome sequencing was also completed at that time, showing two additional VUS in *SLC2A1* and *MT-ND4*.

He reported daily nosebleeds beginning at 4 years of age. He underwent nasal cautery at age 16 years with brief control of nosebleeds, though these have recurred. He had an MRI brain with and without contrast at age 6 years due to possible seizures which showed a few scattered foci of T2 hyperintensity in the subcortical white matter. A repeat brain MRI without contrast at age 12 years and a brain MRI with and without contrast with MRA head at 17 years did not reveal any brain vascular malformations. A saline contrast echocardiogram was negative for intrapulmonary shunting. Familial testing was not pursued because the patient was adopted.

#### 3.1.6. Patient 6

Fourteen-year-old female with celiac disease, attention deficit hyperactivity disorder, and insomnia presented with cyanosis and hypoxia with oxygen saturation of 90% on room air. Contrast echocardiogram revealed intrapulmonary shunting, and a subsequent CTA chest demonstrated several bilateral lung AVMs. She also had a history of frequent nosebleeds since age 6 years, which occurred multiple times per week and for which she had undergone four nasal cautery procedures. Examination revealed two small telangiectasias on the retromolar space and buccal mucosa. Brain MRI with and without contrast and MRA head revealed a closed lip schizencephalic cleft in the medial left frontal lobe with polymicrogyria lining the cleft. There was an associated prominent vein in the adjacent sulcus. She reported around 3–4 migraines per week. A six gene HHT panel revealed a pathogenic *de novo ENG* deletion of exons 9–14 and a paternally inherited missense VUS in *GDF2* (c.41C>T, p.Ser14Phe). The gnomAD allele frequency is 0.000003768, and ClinVar contains a single submission classifying this variant as a VUS. In silico predictions show SIFT: “Not Available”; PolyPhen-2: “Benign”; Align-GVGD: “Not Available”. This variant has not been previously reported in the literature, and there are no functional studies.

Interestingly, both her father and paternal half-brother, who also have the *GDF2* VUS, had significant recurrent nosebleeds (Figure 2c). Her father has two to four nosebleeds per year, some of which last for hours to even days. Her paternal half-brother has experienced nosebleeds since age 12 years and has them at least once a month in his late 20s despite undergoing cautery twice. At the time of this report, neither relative has been examined for telangiectasias or has undergone screening for brain vascular malformations or lung AVMs. The patient’s HHT symptoms are most likely due to her *ENG* pathogenic variant, but given her father and half-brother’s clinically significant epistaxis, a synergistic contribution of her *GDF2* VUS to her symptoms cannot be excluded completely.

#### 3.1.7. Patient 7

Seven-year-old male who presented prenatally due to premature labor at 34 weeks 3 days’ gestation prompting a fetal ultrasound that showed a 2.5 cm vascular malformation. Mother underwent immediate urgent Cesarian section due to risk of rupture with vaginal delivery. A postnatal brain MRI with MRA head showed a vein of Galen malformation. Echocardiogram demonstrated pulmonary hypertension due to left-to-right shunting through the vein of Galen malformation. The infant’s heart failure was managed medically, and embolization was deferred until nine months of age. The patient developed nosebleeds at 2 years of age. A 6 gene HHT panel showed a VUS in *GDF2* c.917G>A p.Gly306Asp. The gnomAD allele frequency is 0.00007249, with the highest frequency in the general African population at 0.09% (71/75,054 alleles). ClinVar has two submissions with conflicting interpretations: one VUS and one likely benign. This variant has not been previously reported in the literature, and there are no functional studies.

While familial testing for the *GDF2* variant has not been completed, it was recommended due to the history of a maternal grandmother with a suspected cerebral vascular malformation and a maternal uncle who died at the age of 28 due to a ruptured cerebral aneurysm (Figure 2d).

### 3.2. Literature Review

Patients with *GDF2* variants from seven other published cohorts are summarized in Table 2. Wooderchak-Donahue et al. performed exome sequencing on a cohort of 38 individuals who met at least two Curaçao criteria for HHT, and three of these patients (unrelated individuals) had heterozygous pathogenic *GDF2* variants (c.254C>T, p.Pro85Leu; c.203G>T, p.Arg68Leu; c.997C>T, p.Arg333Trp) [11]. All presented clinically with epistaxis and cutaneous lesions resembling those found in patients with capillary malformation–arteriovenous malformation syndrome (CM-AVM). Farhan et al. sequenced a cohort of 40 patients who met at least two Curaçao criteria and identified two patients with heterozygous *GDF2* variants (c.1063G>C, p.Glu355Gln; c.1207G>A, p.Val403Ile) and two patients with full gene *GDF2* deletions of at least five megabases [14]. These patients all had clinically significant epistaxis, and 50% had cutaneous telangiectasias.

A recent report identified a family with *GDF2* variants and clinical HHT. The first family member, male, presented in early childhood with epistaxis, telangiectasias, and a diffuse lung AVM without associated pulmonary hypertension. In adulthood, he developed uncompensated cirrhosis without evidence of a hepatic AVM. The sibling and mother also had epistaxis and telangiectasias but no known visceral or brain AVMs [15]. Several of the patients reported had clinical symptoms of HHT and VUS in *GDF2*, which is not surprising given the limited information and more recent discovery of the gene [12,14,15,16,18]. One group reported two individuals with homozygous nonsense variants in the gene, both with skin telangiectasias, but only one with epistaxis and lung AVMs. The other had severe pulmonary arterial hypertension [17]. Thirteen of 15 patients published across the reports had epistaxis, and 12 of 15 patients had skin or mucocutaneous telangiectasias along with various skin findings [12,14,15,16,17,18].

## 4. Discussion

This single-center pediatric case series expands the number of variants reported in *GDF2* and the phenotypic descriptions of children with pathogenic changes and VUS in this gene. We also provide a comparison with other published cohorts of patients with *GDF2* variants and clinical features of HHT. In our cohort, three patients had pathogenic deletions of the *GDF2* gene, and four had missense VUS (one also with pathogenic *ENG* variant); however, only the patient with the missense *GDF2* VUS plus the pathogenic *ENG* variant met more than two Curaçao criteria for HHT. The fact that not even the three patients with pathogenic variants met clinical criteria for “definite” HHT (3 or 4 Curaçao criteria) underscores that the criteria may have decreased sensitivity for HHT diagnosis among children, in whom certain features like nosebleeds and telangiectasias may not become clinically evident until later in life.

Genetic variants are classified in a 5-tier system according to guidelines by the American College of Medical Genetics and Genomics and the Association for Molecular Pathology [21]. Variants are assigned as uncertain, or VUS, when there is insufficient or conflicting evidence to support their classification as pathogenic/likely pathogenic or benign/likely benign. VUS is a frequent assignment for variants in disease-causing genes that have not yet been reported in multiple unrelated probands. To date, there are very few reports of *GDF2*-related HHT and very few reported pathogenic *GDF2* variants. This makes clinical diagnosis and decisions about screening and monitoring difficult in children with potential *GDF2*-related HHT, especially given the known overall poor sensitivity of the Curaçao criteria of merely 68% in children with pathogenic variants in other HHT genes, potentially making the criteria an insufficient tool for HHT diagnosis in the pediatric population [22]. The relatively low sensitivity of the Curaçao criteria makes determination of HHT status especially challenging among children found to have *GDF2* variants as there are relatively few patient reports available.

Given the challenges of clinical diagnostic criteria for HHT in children, our group decided to follow and screen children with *GDF2* VUS and any concerning clinical symptoms, despite not meeting full criteria for the clinical diagnosis of HHT. While this approach has downsides, it avoids the potential catastrophic decision of failing to screen and follow a child with a variant that may be reclassified as pathogenic in the future. Despite almost complete screening for brain and lung AVMs, only two patients in our cohort had a visceral vascular malformation. The only patient with a lung AVM was patient 6, who had a known pathogenic *ENG* variant and for whom any contribution of the *GDF2* VUS is unclear. Patient 7 had a vein of Galen malformation which cannot be definitively associated with his *GDF2* VUS as no lesional tissue was available for somatic genetic testing. It is possible that patients with *GDF2* have fewer signs and symptoms of HHT than children with other HHT variants. For example, children with *GDF2* variants could have less frequent brain and lung AVMs (similar to children with *SMAD4* variants) than children with *ENG* or *ACVRL1* variants [23], but this is unknown. Nonetheless, visceral AVMs or capillary malformations, including in the brain, lungs, and liver, were reported in 8 of the 15 patients from the literature (see Table 2) [12,14,15,16,17,18]. The cases reported in the literature support our current clinical practices of recommending routine surveillance for children with some clinical features of HHT even if they lack a confirmed pathogenic variant. None of the patients in our cohort was identified as having hepatic AVMs, but this may also be due in part to a lack of recommendation for liver AVM screening in asymptomatic children [1]. BMP9 has been demonstrated to help regulate glucose and lipid metabolism in addition to its role in the vascular system, which could explain the formation of hepatic AVMs in some patients with *GDF2* variants [19].

As more patients with *GDF2* variants are identified, it is likely that some VUS will be reclassified as pathogenic, highlighting the need for reevaluation of genetic testing and of reporting cases in the literature to expand what is known about patients with *GDF2* variants, including VUS. This process was notable for our patient 5, whose entire *GDF2* gene was deleted but whose diagnosis was not made until his genetic results were reevaluated many years later after *GDF2*’s function was identified. Additionally, patient 1 in our cohort shared the same *GDF2* c.950G>A missense VUS as a patient identified by Hernandez et al. in 2015, summarized as patient 11 in Table 2 [12]. While our patient with this VUS met only two of four Curaçao criteria, the patient reported by Hernandez et al. met three of four Curaçao criteria, with a history of epistaxis, telangiectasias located on fingers, lips, and ears, and a lung AVM. Likewise, patient 7 in our cohort has a VUS located in the “disordered domain” of the *GDF2* gene, and this same VUS has been reported in a patient with stroke due to a lung AVM, summarized as patient 13 in Table 2 [16]. More research and description of the clinical phenotypic variations seen with *GDF2* variants is needed to determine clinical and screening implications.

When evaluating our cohort for classic Curaçao criteria, we found that our patients overall had a higher frequency of epistaxis compared to other pediatric cohorts [22] but decreased frequencies of telangiectasias (in particular of the lips, fingers, or oral mucosa) and visceral AVMs. Additionally, our cohort had a higher frequency of pulmonary hypertension than cohorts of children with other HHT variants, potentially indicating a unique phenotype for *GDF2*. However, this is speculative given the high degree of phenotypic overlap with patients with other HHT variants and the small size of our cohort.

BMP9 is a ligand in the TGF-β signaling pathway and therefore variations causing structural changes at the molecular interface site may disrupt BMP9-endoglin ligand–receptor binding [14]. This could mean that patients with *GDF2* variants have overlapping phenotypes with patients with *ENG*-related HHT. *ENG*-related HHT is associated with earlier onset of epistaxis as well as higher rates of brain and lung AVMs [17,19,24]. Supporting this possible phenotypic overlap, our report found that all seven patients had nosebleeds which started before adolescence and at a young median age of 6 years, similar to patients with *ENG*-related HHT studied by Hunter et al. [25]. While it is possible that nosebleeds occur overall at younger ages among children with *GDF2* variants, no firm conclusions can be made in such a small cohort.

Pulmonary arterial hypertension was a significant presenting feature in two patients reported in the literature [17,18]. Patient 2 in our cohort presented with pulmonary arterial hypertension, which prompted genetic testing that identified his *GDF2* deletion and secondarily the diagnosis of HHT. Many pulmonary arterial hypertension genetic panels include *GDF2*. It is possible that we are missing HHT features in patients with pulmonary hypertension depending on whether they undergo genetic testing and/or are subsequently evaluated by an HHT expert. Patients with *GDF2*-related pulmonary hypertension may need to be evaluated for brain and lung AVMs.

Unlike the *ENG*-related HHT cohort described in Hunter et al. [25], fewer telangiectasias were identified in our patients with *GDF2* VUS and pathogenic variants. In a cohort aged 16–21 years with non-*GDF2* pathogenic HHT variants, skin telangiectasias were found in only 49% [22], so it may not be surprising that only three of our seven patients had telangiectasias at diagnosis. Three children in our cohort, including one who did not initially have any telangiectasias, developed new telangiectasias over time. It is therefore possible that other children in the cohort who did not have telangiectasias initially might develop these later and thus might meet additional clinical criteria for HHT over time. Telangiectasias in pediatric patients are frequently found on the hands and face, which was common in our cohort, and are less frequently located in the characteristic sites for HHT including the lips, fingers, or oral mucosa [26]. This fact again underscores the challenges of the Curaçao criteria for children with HHT. In contrast, McDonald et al. advocate for modifying the Curaçao criteria to make them more stringent so that the telangiectasia criterion is only met if there are only two or more telangiectasias in characteristic locations (lips, oral cavity, palmar aspect of fingers) and that the epistaxis criterion is only met if there are four or more nosebleeds per year [27]. While children were included in the study by McDonald et al. that may support a modification of the Curaçao criteria [27], many aged 0–18 years had telangiectasias on the dorsum of the hands and other non-characteristic sites. Given that telangiectasias in some people with *GDF2* have been reported to have different appearances than in those with other genetic variants [15], we did not limit reporting of telangiectasias in this pediatric cohort of children with HHT to those with lesions in characteristic areas.

Our series has several limitations. While the inclusion of patients with VUS is important due to the rarity of reported *GDF2* variants, we are not able to attribute the symptoms to these variants definitively. Genome sequencing to investigate deep intronic and non-coding region variants in the other known HHT genes (*ACVRL1*, *ENG*, *SMAD4*) is available as a next tier test, but none of the patients described has undergone genome sequencing. It is important to note the possibility that a deep intronic variant in *ACVRL1*, *ENG*, or *SMAD4* went undetected on standard next generation sequencing panels, as described previously [28], and that the *GDF2* VUS described in our patients are not causing the clinical features of HHT. None of the described patients pursued second tier testing through genome sequencing, which is a limitation of this study. Also, we chose to include our patient with a *GDF2* VUS and a *de novo* pathogenic *ENG* variant because of the interesting possibility of synergy and because both her father and paternal half-brother have clinically significant epistaxis and harbor the same *GDF2* VUS. However, this patient’s phenotype may be mostly, or even all, due to the *ENG* variant. Another limitation is that we were not able to examine all family members and that not all family members had organ AVM screening, so we were not able to characterize relatives’ phenotypes fully. In the future, we will continue to reevaluate the *GDF2* testing of our patients’ VUS to identify any that may be reclassified as benign or pathogenic. Children with *GDF2* VUS and pathogenic variants should have periodic follow-up to determine if they develop additional HHT symptoms.

## 5. Conclusions

In conclusion, our pediatric cohort with *GDF2* pathogenic variants and VUS presents with some features of classic HHT like recurrent epistaxis. The cohort also highlights the difficulty inherent in making a clinical diagnosis of HHT in young children and underscores the importance of ongoing genotype–phenotype correlation in the pediatric HHT population. Although we cannot firmly attribute the clinical signs and symptoms of HHT to the genetic change in patients with VUS in *GDF2*, this cohort serves to expand our understanding of *GDF2* variants. Additional reporting of children with *GDF2* pathogenic variants and VUS will be critical for expanding variant reclassification and confirming decisions regarding imaging surveillance to avoid the most serious complications of HHT.

## Figures and Tables

**Table 1:** Seven patients with *GDF2* variants.

Patient	Age at Clinical Presentation	*GDF2* Genetic Variant	Additional Genetic Variation	Curaçao Criteria (#)	Pulmonary Hypertension	Other Clinical Features
1	3	c.950C>A p.Arg317Gln VUS heterozygous	None	Epistaxis, mucocutaneous telangiectasias (2)	No	None
2	6	Pathogenic deletion (entire coding sequence)	*BMPR1B*: c.1355C>T	Epistaxis, mucocutaneous telangiectasia (single) (1)	Yes	Asthma, heart murmur
3	6	Pathogenic deletion (entire coding sequence)	None	Epistaxis, mucocutaneous telangiectasias (2)	No	Left distal foot truncated and abnormal digits from amniotic band syndrome in utero, dysplastic pulmonary valve
4	9	c.34C>G p.Leu12Val VUS heterozygous	None	Epistaxis (1)	No	Asthma, chronic pulmonary symptoms, pneumothorax
5	4	Pathogenic deletion (entire *GDF2* gene deleted) ^1^	*SLC2A1* c.1387A>T p.Ile463Phe MT-ND4, m.11580C>A p.S274&, 2% heteroplasmy VUS	Epistaxis (1)	No	Possible seizures, behavioral problems, intellectual disability
6	6	c.41C>T p.Ser14Phe VUS heterozygous	*ENG* deletion (Exons 9–14)	Epistaxis, mucocutaneous telangiectasias, lung AVMs (3)	No	ADHD, celiac disease, insomnia, closed lip schizencephaly
7	Prenatal	c.917G>A p.Gly306Asp VUS heterozygous	None	Epistaxis, vein of Galen malformation (2)	Yes (Secondary to shunting)	Epilepsy, headaches, speech delay

# Number of Curaçao criteria met. ^1^ 3.53 Mb deletion includes greater than 20 genes. arr 10q11.22q11.23 (48,300,421–51,832,220) × (Human Genome Build 37, hg19, 2009) including CHAT, ERCC6, NCOA4.

**Table 2:** Patients with clinical HHT and *GDF2* variants in the literature.

Patient (Reference)	Age at First Symptoms (Years)	*GDF2* Genetic Variant Classification	Curaçao Criteria (#) *	First-Degree Family Member HHT Symptoms	Pulmonary Hypertension	Other Clinical Features
1 [14]	37	c.1063G>C p.Glu355Gln missense VUS	Epistaxis, mucocutaneous telangiectasias, liver AVM (4) *	Mother with same VUS—epistaxis, telangiectasias, colonic AVM	No	Diabetes, asthma, paroxysmal SVT, mitral valve regurgitation
2 [14]	9	c.1207G>A p.Val403Ile missense VUS	Epistaxis, brain AVMs (2)	Mother—epistaxis	No	None
				Son—spider veins/possible telangiectasias, epistaxis		
3 [14]	5	5.10 Mb deletion from 10q11.22 to 10q11.23 deletion likely pathogenic	Epistaxis (1)	Unknown—adopted	No	Headaches/migraines, ADHD, SVT
4 [14]	2	5.50 Mb deletion from 10q11.22 to 10q11.23 likely pathogenic	Epistaxis, mucocutaneous telangiectasias, brain capillary malformations, developmental venous anomaly (3)	Not reported	No	Right ventricular hypoplasia, large posterior VSD, secundum ASD
5 [11]	Early childhood	c.254C>T p.Pro85Leu missense pathogenic	Epistaxis, mucocutaneous telangiectasias (2)	Father—autopsy findings suggested HHT but no report available	No	None
				Sibling—epistaxis, stroke at age 43 of unknown cause		
6 [11]	30	c.203G>T p.Arg68Leu missense pathogenic	Epistaxis, mucocutaneous telangiectasias (2)	Father with same VUS—epistaxis	No	Hepatopulmonary syndrome—hepatic findings consistent with likely hepatic VM but not confirmed
				Sister with same VUS—epistaxis		
7 [11]	3	c.997C>T p.Arg333Trp missense VUS	Epistaxis, mucocutaneous telangiectasias (2)	Father—epistaxis	No	None
8 [15] ^±^	Early childhood	c.1282T>C p.Cys428Arg missense VUS	Epistaxis, mucocutaneous telangiectasias, lung AVM (3)	Mother with same VUS—epistaxis, telangiectasias	No	Cirrhosis
				Sister with same VUS—epistaxis, telangiectasias		
9 [15] ^±^	30s	c.1282T>C p.Cys428Arg missense VUS	Epistaxis, mucocutaneous telangiectasias (2) *	Mother with same VUS—epistaxis, telangiectasias	No	None
				Brother with same VUS—epistaxis, telangiectasias, lung AVM		
10 [15] ^±^	Childhood	c.1282T>C p.Cys428Arg missense VUS	Epistaxis, mucocutaneous telangiectasias (2) *	Son with same VUS—epistaxis, telangiectasias, lung AVM	No	None
				Daughter with same VUS—epistaxis, telangiectasias		
11 [12]	Not reported	c.950G>A p.Arg317Gln missense VUS	Epistaxis, mucocutaneous telangiectasias, lung AVM (3)	Sons (2)—epistaxis	No	None
12 [18]	Likely childhood	c.352A>T p.Ile118Phe missense VUS	Epistaxis, mucocutaneous telangiectasias, GI AVMs with anemia (3)	Brother and sister—“HHT-related symptoms” that were not specified, both died of anemia	Yes	None
13 [16]	43	c.871G>A p.Gly291Ser missense VUS	Lung AVM with stroke (1)	No	No	None
14 [17]	3	c.76C>T; p.Gln26Ter nonsense homozygous pathogenic	Skin telangiectasias (1)	Parents heterozygous with no symptoms or clinical signs suggestive of HHT	Yes	Severely dilated right ventricle
15 [17]	9	c.835G>T p.Glu279Ter nonsense homozygous VUS	Epistaxis, skin telangiectasias, lung AVM (3)	Parents heterozygous with no symptoms or clinical signs suggestive of HHT, both parents had negative testing for lung AVMs	No	None

# Number of Curaçao criteria met. * Curaçao criteria for this patient includes family history because a first-degree family member met Curaçao criteria for HHT. ^±^ Cases 8–10 represent a proband, his sister, and his mother.

## Data Availability

The original contributions presented in this study are included in the article. Further inquiries can be directed to the corresponding author.

## Abbreviations

The following abbreviations are used in this manuscript:

HHT	Hereditary hemorrhagic telangiectasia
AVM	Arteriovenous malformation
VUS	Variant of uncertain significance
BMP9	Bone morphogenetic protein
CHOP	Children’s Hospital of Philadelphia
EHR	Electronic health record
ASD	Atrial septal defect
NR	Not reported
SVT	Supraventricular tachycardia
VSD	Ventricular septal defect
VM	Vascular malformation
